# Extracellular Vesicles in Mycobacterial Infections: Their Potential as Molecule Transfer Vectors

**DOI:** 10.3389/fimmu.2019.01929

**Published:** 2019-08-14

**Authors:** Jianjun Wang, Yang Wang, Lijun Tang, Rodolfo C. Garcia

**Affiliations:** ^1^Department of Clinical Laboratory, Affiliated Kunshan Hospital of Jiangsu University, Kunshan, China; ^2^Department of Biochemistry and Molecular Biology, School of Life Science, Central South University, Changsha, China; ^3^Consultant, Beckenham, United Kingdom

**Keywords:** extracellular vesicles, exosomes, mycobacterial disease, macrophages, inflammatory responses, immune responses, biomarkers

## Abstract

Extracellular vesicles are membrane-bound structures released by living cells and present in body fluids. Their composition includes proteins, lipids, carbohydrates, and nucleic acids and are involved in transfers between cells. Extracellular vesicles can deliver molecules to cells and tissues even if distant. As a consequence, they have a role in information transmission and in the modulation of the biological function of recipient cells. Among other things, they are involved in antigen presentation and the induction of secretion events by immune cells. Thus, extracellular vesicles participate in the regulation of immune responses during infections. We will discuss their potential as effectors and disease biomarkers concerning only mycobacterial infections.

## Introduction

It has long been recognized that cells are able to release corpuscles or vesicles of variable size, bounded by an outer bilayer membrane, to the extracellular environment. They are referred to as microparticles, microvesicles, apoptotic bodies, ectosomes, or exosomes (1–3). Extracellular vesicle (EV) release is an evolutionarily conserved phenomenon from bacteria to eukaryotes (4–6). During infections, the EVs released can be pathogen- or host-derived and can therefore contain components from both or just one of them. There is a growing consensus that EVs are important elements in pathogenesis (7, 8). The term exosomes is used for vesicles of 30–150 nm exocytosed from eukaryotic cells after an endosomal invagination process (9). Eukaryotic cells can also release EVs of 100–500 nm by outward budding off the plasma membrane, which have also been named ectosomes or sometimes microparticles (10–13). EVs that bud off from the external membrane of prokaryotes range from 20 up to 1,000 nm (14). With few exceptions, there is a generalized lack of distinction between the different vesicles from host cells and vesicles of bacterial origin in the available literature, which complicates considerably the interpretation of results.

The present review will discuss the effects of EVs on cells and organisms regarding immune responses as well as the feasibility and limitations of EVs as disease biomarkers and vaccine components, in relationship exclusively with mycobacterial infections. The composition and effects of vesicles isolated from infected murine and human host cells, from TB patients' and infected mice sera and from mycobacteria has been analyzed. The fact that the number of publications available involving TB patients is very reduced, together with the common lack of EV type discrimination, does not allow drawing definitive conclusions. The importance of the observations made so far is sufficient, though, to discuss their implications.

### Biogenesis of the Different Extracellular Vesicle Types

Eukaryotic exosomes are generated within the endosomal system through successive stages of endocytosis and multivesicular body (MVB) formation (6, 15, 16). Early endosomes first mature into late endosomes. Invaginations of the late endosomal membrane generate intraluminal vesicles (ILVs) by a process driven by local microdomains and involving the endosomal sorting complexes required for transport (ESCRTs) 0, I, and II but not only (12, 13, 17–19). Post-invagination late endosomes become multivesicular bodies (MVBs) (20). The inward budding process results in small ILVs containing cell cytosol surrounded by a bilayer membrane to which endosomal proteins and receptors are associated (3, 12, 21). MVBs can then either: (1) Fuse with cell lysosomes and follow a degradative path; (2) Merge with the cell plasma membrane and release the ILVs they contain into the extracellular space, where they are referred to as exosomes (22–25); or (3) Merge with phagolysosomes, in the case of infected cells harboring intracellular microorganisms, with the ILVs being then able to interact with phagolysosomal components including the engulfed microorganisms. When phagolysosomes fuse with the plasma membrane and empty their content into the external medium, intraphagosomal ILVs will be released into the external medium. The composition of exosomes can therefore reflect a purely host cell or a host-microorganism mixed origin.

Ectosomes/microparticles are also from eukaryotic origin but they are formed by gradual outward protrusion from the cell plasma membrane through a series of rapid steps. As for exosomes, the generation of ectosomes involves the microdomain ESCRT complexes 0, I, and II (13). The main complex of the ectosome pinching off process is ESCRT III (12).

Regarding prokaryotic cells, EVs (often called microvesicles) are generated from Gram-negative bacteria by outer membrane budding. They encapsulate membrane, periplasmic and cytoplasmic components. Prokaryotic EVs transfer proteins, RNAs, DNAs, and quorum sensing signals to other microbes and to eukaryotic cells (14, 26). In the case of thick-walled mycobacteria, fungi and Gram-positive bacteria, the precise mechanisms of EV generation are far from being fully understood. Formation of EVs from mycobacterial cells has indeed been observed (27, 28). The fact that the mycobacterial plasma membrane is surrounded by a complex peptidoglycan-containing cell wall or mycomembrane and an additional outer capsule means an obstruction regarding the generation and budding of vesicles (29). It is thought that the budding process occurs by means of remodeling enzymes or proteins, similarly to what has been recently reported about EVs from gram-positive *Streptococcus aureus* (30). In the case of *S. aureus*, the generation of EVs is supported by peptides with surfactant activity and by autolysins hydrolyzing highly crosslinked cell wall peptidoglycans. Recent work by White et al. (31) indicates that microvesicle/EV genesis by *M. tuberculosis* involves still uncharacterized components of the regulon Pst/SenX3-RegX3 and is not affected by the ESX-5 type VII secretion system.

### Characteristics of Vesicles From Eukaryotic and Prokaryotic Cells

Exosomes are 30–150 nm in diameter, their buoyant density ranges from 1.13 to 1.19 g/ml and present a cup-shaped morphology under transmission electron microscopy (32). Early descriptions date back to the 1980s, when they were referred to as small membranous vesicles of rat reticulocyte origin in investigations on transferrin recycling (33). These corpuscles were also observed in the supernatant of sheep red blood cells cultured *in vitro* (34). Exosomes can be secreted by a variety of eukaryotic cells, namely dendritic cells (DCs), macrophages, neutrophils, lymphocytes, platelets, mast cells, epithelial cells, neurons, mesenchymal stem cells (MSCs), and cancer cells (35–37). Ectosomes/microparticles are 100–500 nm in diameter and share a number of characteristics with exosomes (11, 12). Extracellular vesicles shed from microbes are on average larger than exosomes (up to 1,000 nm) and are often called microvesicles.

All EV types are membrane-bound corpuscles present in different proportions in cell culture and body fluids. A clear distinction between exosomes and ectosomes from host cells and microvesicles of bacterial origin has been mostly neglected and this has led to mixtures of different vesicles being considered simply “exosomes.” In order to bypass the lack of a proper definition we refer to EVs, exosomes/EVs or ectosomes/microparticles/EVs throughout this review, stating their origin whenever possible from the published information. The term “EV” without a reference to its origin means that it can include vesicles from any origin. The issue of vesicle discrimination is considered again in section Vesicles From Host Cells vs. Vesicles From Mycobacteria. The relevance of EVs stems from their being present in various body fluids: blood, saliva, bronchoalveolar liquid, amniotic fluid, urine, semen, bile, breast milk, cerebrospinal fluid, pleural effusions, and ascites fluid (38, 39).

**Figure 1:**
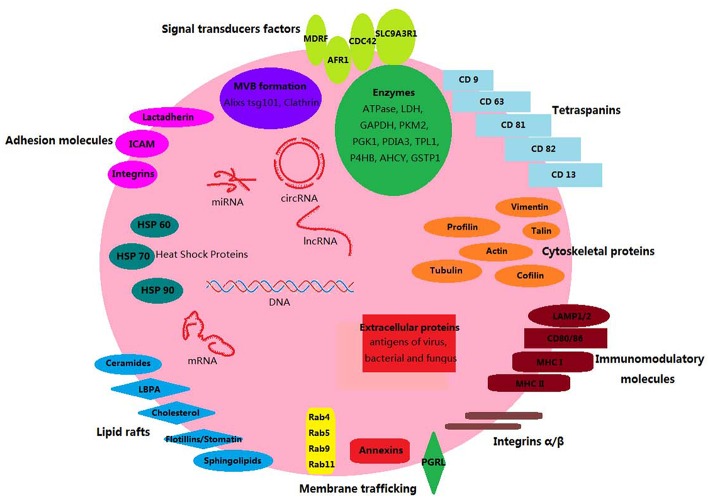
Components of mammalian exosomes. Exosomes are small membrane-bound vesicles. Their lipid bilayer contain typical transmembrane proteins and receptors: tetraspanins (CD9, CD63, CD81, CD82, CD13); signal transduction factors (MDRF, AFR1, CDC42, SLC9A3R1); adhesion molecules (lactadherin, ICAM, integrins); membrane trafficking proteins (annexins, Rabs, PGRL proteins); lipid raft-associated molecules (lbpA, lyn, flotillins/stomatin, cholesterol, sphingolipids); immunomodulatory molecules (LAMP1/2, CD80/86, MHC-I, MHC-II). Luminal proteins have stabilizing, structural, and metabolic functions: HSPs (Hsp60, Hsp70, and Hsp90), cytoskeletal (vimentin, profilin, talin, actin, tubulin, cofilin), enzymes (ATPase, GAPDH, LDH, etc.), MVB biogenesis (alixs, tsg101, clathrin). Other EV components include DNA, mRNAs, miRNAs, cirRNAs, and lncRNAs.

The composition of each EV type depends on the cell of origin and the mechanism of generation. A number of lipid and protein components are common in all the eukaryotic EVs of endosomal origin [Figure 1; (40)]. Endosomal EVs (exosomes) contain transport and fusion-related proteins (flotillin, caveolin-1, annexins, GTPases), tetraspanins (CD9, CD63, CD81), heat shock proteins (HSP60, HSP70, and HSP90), MVB generation proteins (Alix, TSG101), phospholipases (41–44), cytoskeleton and microtubule components, antigen presentation molecules (MHC-I, MHC-II), and signal transduction proteins (CD55, CD59, CD82, Rabs) (42, 45, 46). The composition of EVs from plasma membrane origin (ectosomes, or microparticles) is somewhat different from that of endosomal EVs. For instance, tetraspanins, integrins, and proteoglycans are present in both but are less abundant in ectosome membranes. Instead, the adhesion protein ICAM-1 is present only in exosome membranes, which are rich in other proteins such as receptors, glycoproteins, metalloproteinases, etc. The luminal proteins of both EV types are similar and either anchored mostly by acylation to the membrane, or free in the luminal cavity (low concentrations of cytosolic proteins) (12). The proteins CD63 and CD61 are considered exosome markers, whereas TyA, C1a and CD35, markers of ectosomes (47). Eukaryotic EVs also carry DNA sequences, mRNAs, microRNAs (miRNAs), long intergenic non-coding RNAs (lincRNAs), and circular RNAs (cirRNAs) (48–50), regardless of an endosomal or ectosomal origin.

The transfer of molecules from EVs to target cells can take place via direct fusion of EVs with the plasma membrane of the recipient cell. Fusion occurs when EVs roll over the membrane of the target cell and some of their membrane proteins (probably syncytins) bind to specific target receptors (51). Binding then evolves into fusion by insertion of hydrophobic sequences of EV membrane proteins into the target cell plasma membrane, lipid reorganization, and restructuring, until the EV membrane is finally completely inserted in the target plasma membrane. The content of the EV luminal cargo is then released into the target cell cytoplasm (51). EVs can also transfer their content by being phagocytosed, micropinocytosed or endocytosed (52).

### Intracellular Mycobacteria and EVs

**Figure 2:**
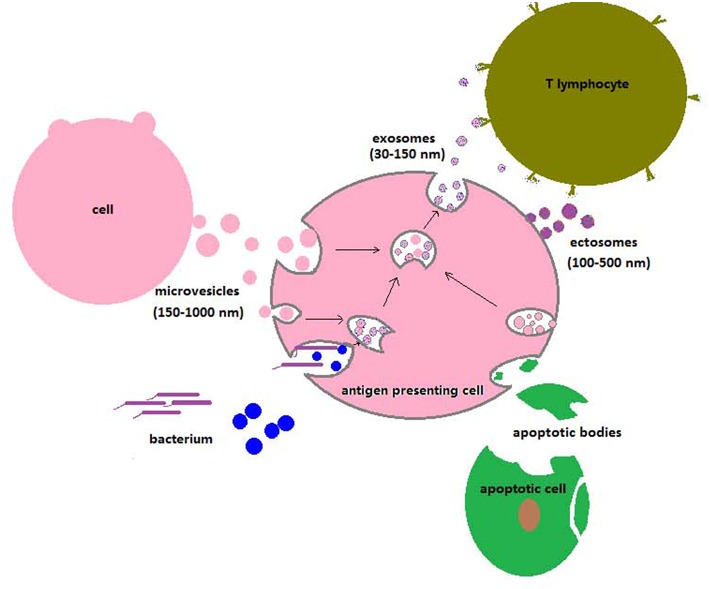
Uptake of particles, vesicle trafficking, and release of EVs to the extracellular space by antigen-presenting cells. The extracellular particles depicted are bacteria, viruses, apoptotic bodies, ectosomes, and exosomes. Ectosomes originate from the plasma membrane, whereas exosomes have MVB origin (see section Biogenesis of the Different Extracellular Vesicle Types in the Text). Invading microorganisms, apoptotic or necrotic cells, and microvesicles of different types are phagocytosed by antigen-presenting cells. Lysosomes fuse with phagocytic vacuoles and generate phagolysosomes, where degradative reactions take place. The phagosome membrane can occasionally rupture, and microorganisms can then translocate to the cell cytosol. Exosomes or ectosomes carrying molecules of microbial origin are released and can interact with T lymphocytes to initiate specific immune responses or with uninfected antigen-presenting cells.

Macrophages are crucial for the immune defenses against mycobacterial infections. Firstly, they are the main host cells. Mycobacteria can proliferate, remain in a quiescent state within them, or be killed, depending on the virulence of the bacterial strains and on immune effector molecules present in the immediate environment of the infected macrophages (53). Secondly, macrophages are also immune effectors themselves. Why does a proportion of the EVs released from infected cells or present in biological fluids of infected organisms likely contain components from the infective agent? Mycobacteria ingested by macrophages, but also by DCs and neutrophils, reside in phagolysosomes formed by fusion of lysosomes with the phagocytic vacuole (54). Proteolysis of bacterial molecules within phagolysosomes generates peptides, some which can be antigenic and/or immunogenic. Degraded/un-degraded bacterial molecules can end up as part of the cargo carried by EVs. In the case of exosomes, this is a consequence of ILV interactions within mycobacterium-containing phagolysosomes. Ectosomes/microparticles of plasma membrane origin can also carry mycobacterial molecules, since mycobacteria can escape from the phagolysosome and become cytosolic (55) or else, mycobacterial components could leak from phagolysosomes. A proportion of the EVs reported to have been shed by macrophages and/or DCs as well as those in sera from infected mice and humans may well be ectosomes in spite of being named exosomes, considering the generalized lack of discrimination between different vesicle types. A close association between EVs and mycobacterial infections has been proposed and hoped for. During an infection, some EVs will derive directly from mycobacteria whereas others, from host cells harboring mycobacteria (macrophages, DCs, neutrophils). EVs could also stem from cells that had taken up apoptotic particles [Figure 2; (56–58)]. Since host cells are undergoing a response to the invasion, a substantial proportion of the EVs generated by them likely carry mycobacterial components (listed on Table 1) and/or pathogenesis-related molecules and are therefore potential effectors of the immune system. Because their cargoes reflect the ongoing process of infection in cells and organisms, EVs become putative biomarkers. In addition, EVs are also hypothetical candidates to deliver molecules with therapeutic purposes, if adequately targeted (69). The present review is circumscribed to mycobacterial infections and we will only refer to EVs generated in that context.

**Table 1:** Mycobacterial components carried by exosomes/EVs.

**Bacterium type**	**Host cells or body fluids**	**Proteins**	**References**
*M. tuberculosis*	Macrophages (*in vitro*)	Lipoprotein, GPL phosphatidylmyoinositol mannosides (PIM)	(17)
*BCG*	BCG (*in vitro*)	LpqH, MPB83, FEIII, LpqL, LppX, LppZ, LpqN, LprA, LprF, LprG, PBP-1, PSTS3, phoS1	(27)
*M. tuberculosis*	*M. tuberculosis* (*in vitro*)	LpqH, FEIII, LprA, LprG	(27)
*M. tuberculosis*	Macrophages (*in vitro*)	Antigen 85C, PckA, GabD1, GabD1, DnaK, LpdC, TB27.3, Cfp29, GltA2,PstS1,TB8.4,LprA,Tal,Cfp17,GlcB,Mpt32,Apa,BfrA,Antigen85B, Rv1906c,KatG,Mpt63,Cfp20/Tpx,Mpt64,HspX,PrcB,GlnA1, AcpM,Cfp2,PepN,Ald,Mpt53,TB22.2,SahH,SapM,GroES,Rv3587c, Mpt51,Antigen 85A,BfrB,ESAT-6	(59)
*M. tuberculosis*	Human serum (*in vivo*)	Antigen 85B, Antigen 85C, Apa, BfrB, GlcB, HspX, KatG, Mpt64	(60)
*M. tuberculosis*	Human serum (*in vivo*)	Cfp2, Mpt32, Mpt64, MrsA, BfrB, Esat-6, GroES, Ag85c, SahH, Ag85a, DnaK, GlnA1, GlcB, AcpM, PpiA, Ag85b, Cfp10, GarA, HspX, MrsA	(61)
*M. tuberculosis*	RAW264.7,HEK293 (*in vitro*)	KatG, HspX, and GroES	(62)
*M. tuberculosis*	*M. tuberculosis* (*in vitro*)	LppX, LpqH (19 kDa lipoprotein), LpqN, LprA, LprF, LprG, PstS1, PstS2, PstS3, HbhA, TatA, Hup, Acn, FbpA (Ag85A), FbpB (Ag85B), FbpC (Ag85C), SodB	(63)
*M. tuberculosis*	Macrophages (*in vitro*)	LpqH (19-kDa lipoprotein)	(64)
*M. tuberculosis*	*M. tuberculosis* (*in vitro*)	LppX, PstS 1,LpqH, Apa,EsxB, Rv1435c, LprA,LprG, MPT63, LprF, EsxA, PstS 2,CysA2, Rv0954, MPT53, Rv1987, Rv0309, MPT64, LpqN, EspC, Cut2, Rv1269c, EspA, TB22.2, Rv1984c, HbhA, TatA, PstS 3, Rv2668, Rv043, Rv1488, Rv2091c, Rv1410c, Hup, MihF, Can, Enolase, MmsA, Rv0315, Gnd2, IlvX, AtpD, Rv0296c, gmdA, Rv0248c, Rv2251, Rv3671c, aldC, icd2, Rv0063, Rv0148, AtsA, MycP3, AtpA, Qor, Ag85A, FadA3, Ag85C, AccA3, Ag85B, MPT51, FadA2, EchA21, FadD13, Rv1544, FadE4, maA4, FadB, SodB, HspX, EphG, Rv3722c, Rv0831c, Rv2159c, ppe41, Rv3099c, Rv3717, Rv3169	(65)
*M. bovis BCG*	Bronchoalveolar lavage fluid of mice (*in vivo*)	19-kDa lipoprotein	(66)
*M. bovis BCG*	Macrophages, mice(*in vitro* and *in vivo*)	Ag85A,HspX	(67)
*M. tuberculosis*	B16 melanoma cells (*in vitro*)	ESAT-6	(68)

## PROTEINS/PEPTIDES/LIPIDs in EVs Released by Mycobacterium-Infected Cells and in Biological Fluids From Mycobacterium-Infected Organisms

As early as 1994, Xu et al. described the ability of *M. tuberculosis*-infected macrophages to traffic bacterial components such as lipoarabinomannans (LAM and ManLAM) out of phagosomes containing bacteria. These components were observed to be transported by distinct intracellular vesicles (70). *In vivo* and *in vitro* studies have subsequently reported that mycobacterial proteins are presented by mycobacterium-infected cells with the involvement of exosomes/EVs or are carried by exosomes/EVs present in cell cultures or body fluids (Table 1). EVs released from mycobacteria-infected macrophages can contain mycobacterial components such as PAMPs (Pathogen-associated Molecular Patterns), which can stimulate the production of pro-inflammatory molecules in recipient macrophages (64, 71). Giri et al. (59) identified by LC-MS/MS 41 mycobacterial proteins in EVs released from *M. tuberculosis*-infected J774 cells and 29 in EVs released from J774 cells treated with *M. tuberculosis* culture filtrate proteins (CFP). Host proteins were also identified. Importantly, EVs from CFP-treated J774 cells were found to promote the activation of macrophages, dendritic cells and naïve T cells *in vivo*. Diaz et al. (72) identified by tandem mass-spectrometry 41 proteins that were more abundant in EVs from *M. tuberculosis-*infected macrophages, out of which 63% were membrane associated. They were all host proteins. Wang et al. (73) analyzed the components of EVs from *M. avium sp. paratuberculosis*-infected macrophages by 2D/MALDI-TOF/TOF. Four macrophage but no *M. avium* proteins were found differentially expressed in the EVs from infected cells. The fact that mycobacterial proteins are not always detected in EVs from mycobacterium-infected cells could be due to levels below detection limits or to a true absence. Since there are different EV subpopulations, it is possible that in some cases the isolation procedure may have selected an EV subpopulation containing only host cell components.

Exosomes isolated from the bronchoalveolar lavage fluid of *M bovis* BCG–infected mice have been found to contain LAM and a 19-kDa mycobacterial, pro-inflammatory lipoprotein (66). Kruh-Garcia et al. (60) identified 20 *M. tuberculosis* proteins in EVs from human serum samples obtained from culture-confirmed active TB culture-positive patients (*n* = 8), employing multiple reaction monitoring mass spectrometry (MRM-MS). Multiple peptides belonged to eight of those proteins, namely Antigen 85B (FrpB), Antigen 85C (FrpC), Apa, BfrB, GlcB, HspX, KatG, and Mpt64. Four proteins are mycobacterial adhesins and the other four are related to bacterial virulence. Moreover, three of the latter are essential for the intracellular survival and host persistence of *M. tuberculosis*. Thus, serum EVs from infected patients show the presence of *M. tuberculosis* proteins involved in the establishment and maintenance of the infection. In 2017, Mehaffy et al. (61) detected 41 *M. tuberculosis* peptides by MRM-MS in EVs from the serum of active TB culture-positive patients from four different geographical locations (*n* = 40). They utilized an optimized data analysis algorithm to reduce the list from 41 to 20 highly significant peptides belonging to the proteins Cfp2, Mpt32, Mpt64, MrsA, BfrB, Esat-6, GroES, Ag85c, SahH, Ag85a, DnaK, GlnA1, GlcB, AcpM, PpiA, Ag85b, Cfp10, GarA, HspX, MrsA, in a disease context. Four of the peptides, corresponding to the proteins Cfp2, Mpt32, Mpt64, and BfrB, were found to be statistically more abundant in individuals with active TB compared with healthy controls. A Cfp2 peptide detected in 92.5% of the patients was singled out as a candidate for diagnosis, considering the heterogeneity, and diversity of the patients studied.

Hare et al. (74) compared the proteomes of plasma membrane EVs (or ectosomes, called microparticles in this case) from *M. tuberculosis*-infected and uninfected human monocytic cells. They found that 68 proteins showed statistically significant differences in their abundance. Forty-two proteins associated with immune function, lysosomal/endosomal maturation, vesicular formation, nucleosomes, and antigen processing were increased in EVs from infected cells. The most prominent were the type I interferon inducible proteins ISG15, IFIT1, IFIT2, and IFIT3. Gonzalez-Cano et al. (75) reported the release of ectosomes containing CD35, Rab5, Rab7, gp91^phox^, phosphatidylserine, and enzymes such as myeloperoxidase and elastase from neutrophils infected with *M. tuberculosis* H37Rv. This observation incorporates another cell of the innate immune system to the picture of EVs as putative effectors.

The investigations described in this section illustrate the potential of mycobacterial proteins carried by EVs as disease biomarkers, although none clearly emerges so far. An increased number of studies will be necessary to establish if and which EVs could participate in the formulation of tuberculosis vaccines (see section EVs Potential as Biomarkers and Vaccination Agents).

## Proteins and Lipids in Mycobacterial EVs

Gram-negative bacteria have long been known to release EVs for cell-to-cell communication with both prokaryotic and eukaryotic cells (14). These bacterial microvesicles can either favor or limit bacterial infections depending on the pathogen in question and on the target cell (76). Microvesicle secretion is a means for cargo sorting and concentrating molecules compared with the situation in the bacteria of origin. It provides a mechanism for bacterial lipids and proteins to traffic while protected from degradation and be then transferred to recipient cells (77).

Mycobacterial EVs have been first isolated from non-pathogenic and pathogenic mycobacteria by Prados-Rosales et al. (27), who reported the identification by LC-MS/MS of 48 proteins in *M tuberculosis* and 66 in *M. bovis* BCG microvesicles. The mycobacterial EVs were enriched in lipoproteins, some of which were virulence-associated. Lipid analysis showed also an enrichment in polar lipids, from which it was inferred that mycobacterial EVs derive from the plasma membrane. The proteins found in *M. tuberculosis* EVs included LpqH (19 kDa lipoprotein), MPB83, LpqL, LppX, LppZ, LpqN, LprA, LprF, LprG, PBP-1, PSTS3, and phoS1 from BCG, and LpqH, FEIII-dicitrate–binding periplasmic lipoprotein, LppX, LprA, and LprG. Some of the mycobacterial lipoproteins and lipids identified are known Toll-like receptor-2 (TLR2) ligands. Notably, EVs were observed to be shed also by intra-phagosomal BCG and *M. tuberculosis*, both *in vitro* and *in vivo* (27). Athman et al. (64) reported the clarifying finding that bone marrow-derived macrophages infected with *M. tuberculosis* H37Ra release two different vesicle EV subpopulations. Only one of the subpopulations contained the mycobacterial lipoprotein LpqH (19 kDa lipoprotein) together with lipomannan and LAM. Lee et al. (63) identified 287 proteins from *M. tuberculosis* EVs by LC-MS/MS, several of which were virulence-associated, namely LppX, LpqH (also known as 19 kDa lipoprotein), LpqN, LprA, LprF, LprG, PstS1, PstS2, PstS3, HbhA, TatA, Hup, Acn, FbpA (Ag85A), FbpB (Ag85B), FbpC (Ag85C), and SodB.

*Mycobacterium tuberculosis* lipoglycans (LAM, lipoarabinomannan, phosphatidylinositol, acylated phosphatidylinositol-dimmannosides, cardiolipin, phosphatidylethanolamine), as determined by mass spectrometry, have been found to be exported *via* microvesicles/EVs secreted either axenically or from mycobacteria inside phagolysosomes, in the latter case being subsequently released into the extracellular medium (27). In this way mycobacterial lipidic molecules can reach and have an effect on target cells, e.g., T cells (78). Iron-deficient conditions (e.g., granulomas) promote a diminished concentration of the cell wall component acyl trehalose in EVs released by *M. tuberculosis* (79), while result in the exclusive presence of the lipidic siderophore mycobactin (80). These observations illustrate the influence of environmental factors on the content of mycobacterial EVs.

The question of how mycobacterial proteins are sorted into EVs has been studied by Smith et al. (62), who determined that ubiquitination of GroES and of the *M. tuberculosis* protein HspX by macrophages is sufficient to direct them to eEVs. Further evidence of ubiquitination as a means to direct proteins to EVs came from the observation that the *M. tuberculosis* proteins Ag85B and ESAT6 are found concentrated in EVs from HEK293 cells when a fusion protein including both proteins is tagged with ubiquitin (81). Moreover, Ag85B/ESAT6-containing EVs were functional and could elicit a T cell response, a subject discussed more extensively in sections Protective/Potentially Protective Responses Induced by EVs Released From Infected Cells and Mycobacteria and EVs Potential as Biomarkers and Vaccination Agents.

## EVs Induce Inflammatory Responses

### Effects of EVs From Host Cells and Mice

The fact that EVs from hosts infected with mycobacteria harbor mycobacterial antigens has prompted the study of their role as inducers of inflammatory responses. Infection with mycobacteria is known to result in macrophage activation and modulation of cytokine secretion, with non-pathogenic microbes being more effective than pathogenic ones (82, 83). EVs released from *M. bovis* BCG-infected macrophages are carriers of mycobacterial cell wall lipids such as lipoarabinomannan (LAM) and phosphatidylinositol mannoside (PIM) (84, 85). These molecules induce the secretion of chemokines and cytokines and promote inflammation (86, 87). The activation of nuclear factor-κB (NF-κB) is correlated with the intracellular survival of mycobacteria. In fact, the mycobacterial proteins Rv2456c, MPT64, PPE37, and Rv3402c activate NF-κB (88). Li et al. (89) found that endothelial cells were activated by EVs derived from *M. tuberculosis*-infected macrophages or mice and containing the *M. tuberculosis* components Ag85 complex and lipoprotein LpqH. These EVs trigger the activation of NF-κB transcription factor and Type 1 interferon pathways to produce CCL2, VCAM1, and TNF-α.

Treatment of mouse bone marrow-derived macrophages with EVs from *M. tuberculosis*-infected RAW 264.7 cells has been reported to enhance expression levels of TNF-α, monocyte chemotactic protein-5 (MCP-5), macrophage inflammatory protein-1α (MIP-1α), MIP-1β, regulated upon activation normal T-cell expressed and secreted (RANTES) and granulocyte colony stimulating factor (G-CSF) (90). In addition, treatment of macrophages with serum-derived EVs from *M. bovis* BCG-infected mice resulted in higher levels of soluble intercellular adhesion molecule-1 (sICAM-1), MIP-2, MIG, MCP5, IP-10, IL-1ra, CXCL13, C5a, MIP1β, RANTES, IL-27, and TNF-α. Giri et al. (59) reported an enhanced production of TNF-α and IL-12 by macrophages and DCs and of TNF-α and IFN-γ by splenocytes following treatment of these cells with EVs released from J774 cells incubated with mycobacterium culture filtrates. Such filtrates contain a number of mycobacterial components that will have been taken up by the J774 cells. IFN-γ is a crucial mediator involved in the JAK-STAT signaling pathway that regulates cellular immunity and inflammatory responses in relationship with defenses against tuberculosis (91, 92).

The *M. tuberculosis* cell wall components 19-kDa lipoprotein and mycolyl-arabinogalactan-peptidoglycan complex (mAGP complex) can induce an inhibition of macrophage responses to IFN-γ. This constitutes a subversion of the immune responses aimed at eradicating the pathogen (93–95). The 19 kDa lipoprotein triggers the generation of IL-1, IL-12p40, and TNF-α by *M. tuberculosis*-infected macrophages through TLR2 ligation. Its important role was highlighted by the work of Stewart et al. (96), who observed that EVs released from macrophages infected with a *M. tuberculosis* mutant lacking a mature 19 kDa lipoprotein fail to induce the secretion of TNF-α and the production of iNOS when incubated with naïve macrophages. Instead, EVs from macrophages harboring wild-type *M. tuberculosis* or a strain complemented with the lspA gene that allows a correct maturation of the 19 kDa lipoprotein are able to promote the secretion of TNF-α and iNOS generation by bone marrow-derived murine macrophages (17, 97). Confirming the results just described, EVs from *M. bovis* BCG-infected J774 cells induced a TLR- and myeloid differentiation factor 88-dependent pro-inflammatory response in uninfected macrophages (66). Similarly, EVs from macrophages infected with either BCG or *M. tuberculosis* administered intranasally into mice stimulated TNF-α and IL-12 production in the lungs. Moreover, EVs isolated from the bronchoalveolar lavage fluid (BALF) of *M. bovis* BCG-infected mice and carrying mycobacterial lipoarabinomannan and 19 kDa lipoprotein can induce TNF-α production in naïve macrophages (66).

An interesting study has recently analyzed the effects of interfering with the last step of exosome biogenesis by genetic manipulation. Since the protein Rab27a is implicated as a key regulator of MVB fusion with the plasma membrane (98, 99), EVs from Rab27a-deficient and wild-type C57BL/6 mice were compared regarding their abundance, composition and effects. Macrophages from *M. tuberculosis*-infected Rab27a-deficient mice were found to release only 20% of the EVs that infected wild-type macrophages do (67). In support of this observation, infected Rab27a-deficient mice showed a ~30% lower serum EV concentration and a ~100% higher bacterial load compared with wild-type animals. These results suggest that, at day 40 after mycobacterial infection, 2/3 of the EVs in mouse serum are exosomes (i.e., EVs of endosomal origin), with EVs being particles isolated from 220 nm filtrates by differential centrifugation (10,000–100,000xg). Moreover, treatment of bone marrow-derived macrophages with EVs from the serum of *M. tuberculosis*-infected wild-type C, 57BL/6 mice induced the production of chemokine C-C motif ligand1 (CCL1), IFN-γ, RANTES, MIP-2, IL-1R, and TNF-α to levels remarkably higher than those induced by treatment with EVs from infected Rab27a-deficient mice. This indicated that EVs from Rab27a-deficient mice are less pro-inflammatory (67).

*Mycobacterium avium*, while less pathogenic than *M. tuberculosis*, shares a number of features regarding the macrophage responses it elicits. EVs from *M. avium*-infected THP-1 macrophages, which contain the *M. avium* proteins ESAT-6, MPT63, SodA, MPT51, and antigen 85-C, can induce macrophages to produce the pro-inflammatory cytokines IL-6, IL-8, IL-10, IFN-γ, and TNF-α as well as to express cell surface molecules related to acquired immunity such as CD40, CD80, CD81, CD86, CD195, and HLA-DR (100). This indicated that the macrophage response to EVs containing *M. avium* proteins is similar to that to *M. avium* itself, as reported by Gidon et al. (101). As to the signaling mechanisms involved, the secretion of TNFα, IL-6, and IL-10 by human macrophages upon infection with *M. avium* is mediated by TLR/MyD88 (101). The myeloid differentiation primary response gene 88 (MyD88) is a TLR-adaptor protein that participates in innate and acquired immune responses to *M. tuberculosis*. It is involved in the downstream signaling of all TLRs except for TLR3 and in preventing excessive inflammation and cellular damage in the lung (102). Regarding the mycobacterial components that trigger TLR signaling and inflammation, it has been reported that ManLAM binding to DC-SIGN on human DCs activates the serine/threonine kinase Raf-1, leading to phosphorylation and acetylation of p65, the activating subunit of NF-kB (103).

Vesicles released by eukaryotic cells can have a plasma membrane as opposed to endosomal origin. Walters et al. (104) isolated and identified spherical, CD45+ microparticles (MPs) of 100–1,000 nm which are not exosomes but ectosomes, based on their size and EM morphology. MPs are released from *M. bovis* BCG- and *M. tuberculosis*-infected macrophages as well as present in the serum of aerosol-infected mice. These MPs induce neutrophil, macrophage, and dendritic cell recruitment at the site of injection into uninfected mice. They enhance the release of proinflammatory cytokines and chemokines by naive macrophages and favor the egress of cells to the site of *M. tuberculosis* infection in the lung by disrupting respiratory epithelial cell monolayers. *M. tuberculosis*–derived MPs are able to activate *M. tuberculosis*–specific CD4+ T cells *in vivo* and *in vitro*, an indication that they carry mycobacterial antigens. Hare et al. (74) studied the effect of ectosomes, which they call MPs, derived from *M. tuberculosis*-infected and uninfected human monocytic cells. Treatment of uninfected monocytic cells with MPs from infected monocytes induced the increased release of the proinflammatory cytokines IL-8, MIP-1α, and IP-10. Alvarez-Jimenez et al. (105) evaluated the effect of ectosomes from neutrophils infected *in vitro* with *M. tuberculosis*. When macrophages were treated with such EVs for 24 h, an increase in the secretion of TNF-α, IL-6, and IL-10 was observed. Additionally, the infected neutrophil-derived ectosomes caused a reduction in the intracellular bacterial load of *M. tuberculosis*-infected macrophages. This could be due to a concomitant increase in the superoxide generation rate which, since superoxide stimulates autophagy, would be responsible for the enhanced mycobacterial killing.

In conclusion, the results of the *in-vivo* and *in-vitro* experiments described in the present section support a role for EVs in the pathology of mycobacterial infections. EVs can induce inflammatory responses on account of the components they carry.

### Effects of EVs Released by Mycobacteria

Experiments on the role of mycobacterial vesicles in infections were first reported by Prados-Rosales et al. (27). These authors observed that microvesicles were shed from intracellular *M. bovis* and *M. tuberculosis* and trafficked to other locations within the host macrophages. Such microvesicles are enriched in mycobacterial lipoproteins known to be TLR2 ligands (LpqH, LprG, PhoS1) and glycolipids (LAM, phosphatidylinositol mannosides). Not surprisingly, they induce murine macrophages to secrete a range of cytokines (IL-1β, IL-6, IL-12, IL-10, TNF, CXCL1, and MIP-1α/CCL3) and inflammatory mediators, mostly in a TLR2-dependent manner (27). The induction of IL-10 secretion by EVs from pathogenic mycobacteria is detrimental concerning mycobacterial infections, since IL-10 is a down-regulator of macrophage function (28). Thus, the fact that EVs can exert simultaneously pro- and anti-inflammatory effects points to a dual effect the balance of which might be influenced by other factors during infections, for instance environmental conditions. Another detrimental effect of mycobacterial EVs is that they can impair the control of experimental infections when injected 2 weeks before exposure to *M. tuberculosis* H37Rv aerosols, as shown by an increased occurrence of granulomas, granulomatous inflammation and bacilli dissemination (27). This finding is against a role for EVs released from mycobacteria in vaccine development. It is important to consider here that while TLR-mediated events generally promote immunity, a prolonged period of TLR2 agonism during tubercular infections (e.g., within a granuloma) results in the secretion of immunosuppressive cytokines (e.g., IL-10) and the down-regulation of MHC-II antigen presentation (106, 107). The host can then mount CD4+ T cell responses to limit the proliferation of bacilli within the granuloma without eliminating them, i.e., a situation of latent infection. Dual effects of EVs have also been shown by Jurkoshek et al. (77), who reported that *M. tuberculosis* EVs do inhibit macrophage and T cell functions, but induce MHC-II antigen presentation by dendritic cells.

Intratracheal administration of EVs released by *M. tuberculosis* causes inflammation in the lungs of WT but not TLR2-deficient mice, in agreement with EVs acting through the TLR2 ligands they carry. One major TLR2 agonist carried by *M. tuberculosis* EVs is the lipoprotein LpqH. Mycobacterial strains deficient in the gene rv0431 (“vesiculogenesis and immune response regulator” or virR), which regulates mycobacterial EV formation, exhibit an hypervesiculating phenotype. The increased amounts of EVs produced by virR-deficient *M. tuberculosis*, acting through TLR2, act on human primary macrophages to stimulate the secretion of TNFα and IL-6. This means that virR somehow restricts the generation of EVs, so limiting the extent of TNFα and IL-6 secretion by the macrophages the EVs interact with (108).

Altogether, a conflictive picture emerges about the effects of mycobacterial EVs on hosts due to an unpredictable balance between desirable and immune evasive effects (77). Future studies need to address the problem of the composition of mycobacterial vesicles, its variability as a function of biological conditions and the possibility of custom bioengineering them.

## Protective/Potentially Protective Responses Induced by EVs Released From Infected Cells and Mycobacteria

A protective anti-mycobacterial immune response necessitates the participation of CD4+ and CD8+ T memory cells (109, 110). Tuberculosis patients mount a Th1 response to mycobacterial infections, shown by the presence in blood and lungs of CD8+ and CD4+ T cells that respond specifically to mycobacterial antigens by replicating and secreting IFN-γ and other Th1 cytokines (111–113). Immune activation occurs typically in the lymph nodes but is also observed in granulomas. Granulomatous mycobacteria are sequestered within macrophages physically removed from the antigen processing machinery leading to an acquired immune response. Substantial CD8+ T cell activation is still observed within granulomas (114–116). Infected macrophages are poor presenters of mycobacterial antigens to both CD8+ and CD4+ T cells, thus the acquired immune response within granulomas is likely the result of alternative mechanisms of antigen presentation (117–119). One such mechanism is the transfer of molecules *via* EVs that contain mycobacterial components and immune-related molecules.

EVs released by *M. tuberculosis* induce murine DCs to increase substantially the expression of MHC-I, MHC-II, and CD86, all involved in antigen presentation (77). This demonstrates that EV components favor an acquired immunity by inducing DC maturation. Furthermore, DCs co-cultured with Ag85b-specific CD4+ T cell hybridoma cells and *M. tuberculosis* EVs are able to activate T cells, detected by IL-2 production. This strongly suggests that EVs released from *M. tuberculosis*-infected cells are able to transfer extracellular antigens such as Ag85b to lymph node DCs and prime CD4+ T cells (77).

Proteins of the PE_PGRS family are mycobacterial proteins carried by EVs that participate in immunological events. PE_PGRS and PE are products of the PE_PGRS gene and are found associated to the mycobacterial cell wall (120). They are able to generate a humoral immune response (121) and influence the interaction of mycobacteria with other cells (122). Expression of PE_PGRS in *M. smegmatis* results in a better survival rate of this bacterium after intraperitoneally administration into mice as well as within macrophages, in the latter case inducing the production of greater levels of TNF-α and progression to necrosis (123). Proteins of the PE family are extracellularly released *via* EVs from *M. tuberculosis*-infected bone marrow-derived dendritic cells and macrophages. Moreover, such proteins were detected in T cells co-cultured with infected dendritic cells (124). The latter observation is consistent with the prior release of EVs transporting these mycobacterial proteins from infected cells. Delivery of such molecules to T cells would start the path to immunogenicity against them.

Intranasal administration of exosomes from *M. bovis* BCG-infected macrophages has been found to induce splenic CD4+ and CD8+ memory cells in BCG-sensitized mice. The fact that CD8+T cells produced IFN-γ upon re-stimulation with BCG antigens indicated that antigens present on exosomes were presented through the MHC-I pathway (125). A further example of the contribution of EVs released from *M. tuberculosis*-infected cells to T cell responses has been provided by Ramachandra et al. (126). These authors show that, upon infection of macrophages with *M. tuberculosis* or *M. bovis* BCG, exosomes and plasma membrane-derived microvesicles (ectosomes) bearing MHC-II were released. These organelles were both able to present peptide-MHC-II complexes to and stimulate T hybridoma cells, i.e., a step toward the onset of antimicrobial actions (126). Smith et al. analyzed the contribution of EVs to a T cell response employing wild type and Rab27a-deficient mice infected intranasally with *M. bovis BCG* containing an Ag85A-DsRed marker antigen (67). Rab 27a is involved in exosome biogenesis (98, 99). EVs isolated from BMMs of the Rab27a-deficient mice were found to carry only 20% of the marker antigen that exosomes from wild type BMMs express. Accordingly, the number of splenic and lung T cells that produced IFN-γ in Rab27a-deficient mice upon antigen stimulation was 10–20% of that in wild type animals. Studies by Giri et al. (59) showed that CD4+ and CD8+ cells isolated from spleens, lungs and mediastinal lymph nodes of mice immunized with EVs released from macrophages incubated with *M. tuberculosis* culture filtrate proteins did indeed proliferate in response to those proteins. Moreover, IFN-γ secretion was simultaneously stimulated. Earlier work by Giri et al. had reported the induction of memory T cells by EVs, in mice (125) (see section EVs Potential as Biomarkers and Vaccination Agents). A clear protective effect of EVs was reported by Prados-Rosales et al. (127) in a mouse model (section EVs Potential as Biomarkers and Vaccination Agents).

The primary role of IFN-γ in host responses to mycobacteria is to enhance the ability of macrophages to control infection (128). IFN-γ has been reported to facilitate antigen processing and presentation to CD4+T cells through the up-regulation of MHC-II expression (129), but this effect is inhibited in *M. tuberculosis*-infected macrophages (130). Nevertheless, IFN-γ induces autophagy and this favors *M. tuberculosis* killing due to the activation of the IFN-γ inducible, immunity-related p47 GTPase Irgm1 also known as LRG-47 (131, 132). EVs from *M. tuberculosis*-infected macrophages reproduce the interference of mycobacterial infection with the IFN-γ induced up-regulation of MHC-II and other genes. In fact, the expression of IFN-γ induced MHC-II and CD64 by bone marrow-derived macrophages was inhibited by EVs from *M. tuberculosis* H37Rv-infected macrophages (133). Such inhibition was observed to be completely dependent on TLR2 and MyD88 and involve the down-regulation of a MHC-II trans-activator molecule. Athman et al. (134) reported that microvesicles originated from intracellular *M. tuberculosis* and then released from the host macrophages inhibit IL-2 generation by CD4+ T cells and reduce T cell proliferation. The released EVs, of bacterial origin, contain lipoglycans and lipoproteins that modulate TLR2-dependent cytokine generation and MHC-II expression (95, 106, 135–137). In summary, mycobacterial molecules aimed at inhibiting phagosome maturation can be carried by EVs, therefore expanding the range of action of the pathogenic microorganisms.

EVs can also influence cellular displacements related to infection/inflammation. Mouse bone marrow macrophages treated with exosomes released from *M. tuberculosis*-infected RAW264.7 cells released increased amounts of a number of chemokines that attracted splenic macrophages, neutrophils and T cells in trans-migration assays (90). When injected intra-nasally, these EVs promoted an influx of CD11b+ cells to the lungs *in vivo*. Hollow fibers containing exosome-treated macrophages and then implanted subcutaneously resulted in increased macrophage infiltration around the fibers, in agreement with chemokines diffusing from inside the fibers to the surrounding area and acting as cell attractants (90).

## EVs Potential as Biomarkers and Vaccination Agents

Exosome/EV research has expanded the knowledge about microorganism-host interactions through the realization that invading microorganisms can extend their range of action beyond their actual physical location within infected organs. They do so through the release of tiny vesicles carrying molecules taken from the cell of origin as well as from hosted microorganisms. These molecules can be antigenic and/or modulate physiological mechanisms to fight infections (138). Consequently, new possibilities about diagnostic and therapeutic strategies have been opened in relationship with a number of infectious diseases including TB (139).

EVs are widely present in biological fluids and are stable in the circulation, so preserving proteins from degradation. EVs persist through the course of infections, increase their numbers in blood during inflammation and can bind to and be endocytosed by APCs thanks to the presence of adhesion molecules (140). These characteristics make EVs excellent candidates as biomarkers and potential vaccination agents (7, 22, 141).

### EV Isolation Methods

Rapid and relatively simple methods of vesicle purification and analysis are essential to conduct *in-vivo* experimentation and, most importantly, animal and clinical trials (142). Although EV isolation from serum and other biological fluids is relatively straightforward, separation and discrimination of different vesicle types is not well defined. Initial difficulties to isolate vesicles in relatively large amounts despite their low concentration in biological fluids have been gradually overcome thanks to recent technology developments. EVs can be purified from infected cells and organisms using methods described in detail by Ko et al. (143), which we will briefly outline here. Differential centrifugation is based on the initial elimination of large size particles of high sedimentation velocity by low speed centrifugation. The resulting supernatants are then subjected to centrifugation steps of increasing speed, at the end of which a pellet enriched in vesicles is obtained. Isopycnic separation on sucrose gradients or OptiPrep™ is based on particle densities (144). It is a convenient technique except in the presence of virus particles which might have a similar density. A more recent nanotechnological procedure utilizes a ciliated nanowire-on-micropillar structure that traps vesicles, which are subsequently released. Acoustic sorting is another novel method based on the movement of particles in an applied acoustic field, where radiation forces proportional to particle volumes drive the faster migration of larger particles. This results in objects of different size separating from each other in different laminar flows (145). Precipitation with highly hydroxylated, water excluding polymers such as polyethylene glycol, dextrans, and polyvinyls is a method traditionally used to isolate viruses which has been adapted to exosome purification (146) and is the basis of the commercial kits ExoQuick (System Biosciences) and Total Exosomes Isolation Kit (Life Technologies). Immuno-affinity-based methods rely on the presence of specific proteins on vesicle surfaces, which allows the use of antibodies against those proteins as trapping agents. Employing this technique, EV subpopulations expressing different markers can be separated. The isolation procedure involves the use of magnetic activated cell sorting (MACS, Miltenyi Biotec) columns. Columns consist of magnetic nanoparticles to which antibodies with affinity for protein/s present on the surface of EVs have been coupled (147). The vesicles will then remain trapped, whereas other components of the fluid will flow through. Vesicles from plasma, cell culture supernatants or body fluids like urine or ascites are usually trapped using anti-tetraspanins (CD9, CD63, and CD81) (142). This procedure will exclude vesicles lacking tetraspanins on their surface, among which those of mycobacterial origin are likely to be included. Pre-purified EV preparations can be used as the starting material to isolate specific subpopulations, e.g., vesicles expressing antigenic mycobacterial proteins (Ag85, ESAT6, Rv2660c) that are better candidates to elicit immunity in recipient organisms. Selective binding of vesicles to antibody-coated, miniaturized microfluidic chips is an immuno-affinity-based method offering increased sensitivities and reduced costs and suitable for medical diagnostics and blood analysis. Small sample volumes can be quickly processed in microfluidic devices requiring minimal amounts of reagents (148).

Isolation procedures will have to expand and take advantage of the sedimentation velocity of different vesicle types or the presence of markers or marker sets for separative flow cytometry and/or immune-affinity based methods.

### Vaccine and Biomarker Perspectives

The protein composition of vesicles isolated from infected cells, mycobacterial cultures or body fluids can be studied by means of mass spectrometry analysis, with further Western blot validation whenever possible. A statistical treatment of quantified proteomic and imaging data can lead to the definition of prospective diagnosis and prognosis biomarkers. Regarding markers, those related to early stages of tubercular disease should be aimed at (149). Proteins identified up to date, some of which could turn out to be of diagnostic value, have been discussed in sections Proteins/Peptides in EVs Released by Mycobacterium-Infected Cells and in Biological Fluids From Mycobacterium-Infected Organisms and Proteins and Lipids in Mycobacterial EVs and are listed in Table 1.

Ziegenbalg et al. (65) evaluated the human antibody responses to EVs from BCG and *M. tuberculosis* by ELISA and immunoblotting, with the aim of identifying potential TB biomarkers. The reactivity of 28 sera from culture-positive TB patients and 16 from controls were examined. EVs from both BCG and M. tuberculosis were strongly immunogenic for TB patients but not in controls. Three proteins of ~36, 25, and 23 kDa were recognized by sera from 25/28 TB patients and 0/16 controls. These results indicate that antibody responses to proteins present in EVs from pathogenic mycobacteria may constitute a novel TB biomarker signature with diagnostic implications.

Apart from vesicle isolation and analysis, the feasibility of hypothetical EV-based or -supplemented vaccines depends on animal experimentation and clinical trials. The use of vesicles for vaccination purposes has been already considered and developed regarding cancer vaccines (150, 151). Regarding mycobacterial infections, EVs released from infected macrophages have been reported to stimulate both CD4+ and CD8+ splenic T cells isolated from mycobacteria-sensitized mice (125). These EVs contain MHC I and II and costimulatory molecules for antigen presentation but in order to achieve a greater stimulation of T cells, EVs had to be incubated with antigen presenting cells. Furthermore, EVs from *M. bovis* BCG- and *M. tuberculosis*-infected macrophages stimulated bone marrow-derived dendritic cells. Intranasal challenge of mice with EVs from *M. bovis* BCG-infected macrophages induced the generation of memory CD4+ and CD8+ T cells. Additionally, the T cells secreted IFN-γ when re-stimulated with BCG antigens, *in vitro* (125). EVs are more efficient molecule presenters than the cells of origin, likely due to a relative higher concentration of immunity relevant mycobacterial molecules. It is not yet clear whether some specific component/s or the combination of several of them are responsible for the effects observed (125, 152). Vesicles from CFP-treated macrophages, which contain mycobacterial antigenic proteins, reportedly induced inflammation as well as the activation of DCs and antigen-specific T cells after intranasal administration to mice (59). CD44^hi^, CD62L^low^, CD4+, and CD8+ T cells, indicative of effector memory cells, were found in lungs, spleen, and mediastinal lymph nodes. This result suggests that a vaccination-like treatment of mice with exosomes isolated from CFP-treated macrophages can induce a profile of T cell activation often associated with *M. tuberculosis* control (i.e., IFN-γ production and effector memory cells) (59). The fact that CFP-exosomes/vesicles can activate T cells *in vivo* in the absence of added adjuvants is particularly important and strongly suggests a vaccine-like effect. However, protection against tubercular infection was not actually demonstrated. A later publication of the same team did show that exosomes/vesicles from CFP-treated macrophages actually primed a protective immune response in mice challenged with a low-dose *M. tuberculosis* aerosol, and boosted a prior BCG immunization (153). Crucial work by Prados-Rosales et al. (127) showed that EVs from BCG or *M. tuberculosis* injected to mice elicit a humoral/cellular response directed to bacterial membrane and cell wall components. *M. tuberculosis*-derived EVs were able to protect against a *M. tuberculosis* aerosol challenge to the same level as live BCG immunization does. Additionally, EVs from *M. tuberculosis* boosted the effect of the BCG vaccine. Therefore, bacterial EVs are able to function as a vaccine in the absence of adjuvants, probably due to the fact that these vesicles can present immunogenic components in the lipidic context of a bacterial membrane.

An interesting concept in vesicle-based vaccines is the introduction of additional molecules aimed at enhancing antigenicity. This has been performed by Koyama et al. (68), who isolated exosomes from a melanoma cell line transfected with a plasmid encoding the mycobacterial protein ESAT-6. The modified exosomes evoked a clear cellular immunity against both ESAT-6 and the tumor cells. Cheng et al. (81) reported that a C-terminal fusion of ubiquitin to the *M. tuberculosis* proteins Ag85B and ESAT6 served as an efficient delivery sequence into EVs when expressed in HEK-293 cells. The concentration of Ag85B and ESAT6 in EVs was found to be significantly increased. When such EVs were used for immunization, a direct correlation was observed between the amount of fusion protein within the vesicles and the number of Ag85B- and ESAT6-specific INF-γ-secreting T lymphocytes present in lungs and spleens. This indicated that exosomes containing a recombinant antigen can elicit a T cell response, hence could be developed as potential vaccines.

Although knowledge about mycobacterial infection-derived EVs has undoubtedly advanced, their possible use as vaccines will need additional research to reach the stage of clinical trials. Important questions still to be answered include the composition variability of EVs, the variability parameters and the introduction of immunogenicity-enhancing molecules in EVs.

## Vesicles From Host Cells vs. Vesicles From Mycobacteria

An important issue to be clarified concerns the actual origin of EVs isolated either from the culture medium of mycobacterium-infected cells or from body fluids. Its importance, already mentioned in this review, deserves a more detailed discussion and a summary of findings. EVs could be originated, alternatively or simultaneously, from: (1) Host cells, through an endosomal process (exosomes); (2) Host cells, by outward budding of the plasma membrane (ectosomes) (11, 74, 104); (3) Mycobacteria inside phagosomes, from which microvesicles bud off to subsequently make their way to the extracellular space (27) by mechanisms yet to be established, or (4) Mycobacteria that have translocated from the phagolysosome to the cytosol, after which they could shed microvesicles that manage to traffic to the extracellular space. Complete evidence about point 4 is not yet available and only the translocation of mycobacteria from phagosome to cytosol has been reported (55).

The existence of different vesicle types was nicely evidenced by Athman et al. (64), who reported that bone marrow-derived macrophages infected with *M. tuberculosis* H37Ra release two vesicle subpopulations: one of them contains mycobacterial molecules (lipoglycans, lipoproteins) whereas the other exhibits exosome markers such as CD9 and CD63. These vesicles appeared to be of similar size in both subpopulations and could be separated due to their different equilibrium density on sucrose gradients. Moreover, the release of the vesicle subpopulation containing mycobacterial components was found to be dependent on the viability of intracellular mycobacteria, which implicates bacterial mechanisms that should be active. A further discrimination is that between exosomes and ectosomes. Host cells could in fact generate both simultaneously but their separation presents problems due to considerable overlaps in size, density and composition.

In conclusion, the different types of EVs (exosomes, EVs of bacterial origin, ectosomes) have been seldom distinguished in the published literature until recently. Investigations have been mostly focused on the effects of EVs on immune and inflammatory responses but not on to the actual origin of the vesicles themselves. Protein profiles of purified EVs obtained from *in-vivo* and *in-vitro* mycobacterial infections will be necessary to characterize the different EV types and be able to interpret their effects.

## Outlook

Research on extracellular vesicles in relationship with *M. tuberculosis* infection is still at an early stage. The following findings link EVs and mycobacterial infections: (a) A good correlation between bacterial load and serum concentration of exosomes/EVs/ectosomes, observed in *M. bovis* BCG- and *M. tuberculosis*-infected mice (90, 104); (b) A variability in the composition of EVs as a function of infection times, inferred from the extent of the responses elicited (90); (c) An enrichment of mycobacterial effector molecules in EVs; and (d) A more efficient presentation of effector molecules compared with the cells of origin (59). The latter could well be the result of a proportion of these vesicles being of bacterial origin. To further clarify the points mentioned above, detailed studies on the molecular composition (lipids, proteins) of EVs from uninfected and infected host cells and organisms will have to be performed at different infection times and after administration of therapeutic agents. All this implies reproducible separations of host cell EVs from bacterial EVs and careful characterizations. Of particular relevance will be the evaluation of the composition of EVs from biological fluids of mice and humans (serum, sputum, bronchoalveolar lavages) in relationship with tubercular disease stages and as a function of responses to anti-tubercular treatments. Further studies are also needed on vesicle biogenesis and cargo sorting, for both host and bacterial vesicles. Future aims should be defining which EVs (source, characteristics) could be employed for diagnostic, prognostic, and therapeutic purposes, and whether the insertion of selected molecules can improve immunogenicity or target cell responses, in relationship with vaccine development.

## Author Contributions

LT and RG conceived, designed, discussed the work, and revised the manuscript. JW, YW, and LT wrote and edited the manuscript. All authors read and approved the final version.

### Conflict of Interest Statement

The authors declare that the research was conducted in the absence of any commercial or financial relationships that could be construed as a potential conflict of interest.
